# Delivering exceptionally safe transitions of care to older people: a qualitative study of multidisciplinary staff perspectives

**DOI:** 10.1186/s12913-020-05641-4

**Published:** 2020-08-24

**Authors:** Ruth Baxter, Rosemary Shannon, Jenni Murray, Jane K. O’Hara, Laura Sheard, Alison Cracknell, Rebecca Lawton

**Affiliations:** 1 Yorkshire Quality and Safety Research Group, Bradford Institute for Health Research, Bradford, UK; 2 School of Healthcare, University of Leeds, Leeds, UK; 3 York Trials Unit, University of York, York, UK; 4 Leeds Centre for Older People’s Medicine, Leeds Teaching Hospitals NHS Trust, Leeds, UK; 5 School of Psychology, University of Leeds, Leeds, UK

**Keywords:** Patient safety, Transitions of care, Hospital discharge, Elderly care, Health care professionals, Qualitative, Focus groups, Positive deviance

## Abstract

**Background:**

Transitions of care are often risky, particularly for older people, and shorter hospital stays mean that patients can go home with ongoing care needs. Most previous research has focused on fundamental system flaws, however, care generally goes right far more often than it goes wrong. We explored staff perceptions of how high performing general practice and hospital specialty teams deliver safe transitional care to older people as they transition from hospital to home.

**Methods:**

We conducted a qualitative study in six general practices and four hospital specialties that demonstrated exceptionally low or reducing readmission rates over time. Data were also collected across four community teams that worked into or with these high-performing teams. In total, 157 multidisciplinary staff participated in semi-structured focus groups or interviews and 9 meetings relating to discharge were observed. A pen portrait approach was used to explore how teams across a variety of different contexts support successful transitions and overcome challenges faced in their daily roles.

**Results:**

Across healthcare contexts, staff perceived three key themes to facilitate safe transitions of care: knowing the patient, knowing each other, and bridging gaps in the system. Transitions appeared to be safest when all three themes were in place. However, staff faced various challenges in doing these three things particularly when crossing boundaries between settings. Due to pressures and constraints, staff generally felt they were only able to attempt to overcome these challenges when delivering care to patients with particularly complex transitional care needs.

**Conclusions:**

It is hypothesised that exceptionally safe transitions of care may be delivered to patients who have particularly complex health and/or social care needs. In these situations, staff attempt to know the patient, they exploit existing relationships across care settings, and act to bridge gaps in the system. Systematically reinforcing such enablers may improve the delivery of safe transitional care to a wider range of patients.

**Trial registration:**

The study was registered on the UK Clinical Research Network Study Portfolio (references 35272 and 36174).

## Background

Transitions of care from hospital to home are risky. One in five patients experience an adverse event during this transition, 62% of which could be prevented or minimised [1]. In the United Kingdom (UK), readmission rates are used as an indicator of quality and they have risen by 22.8% since 2012/2013 [2]. Although not all readmissions represent poor quality care, around 30% are considered to be potentially avoidable [3–5] and this figure is thought to increase if readmissions due to deconditioning and post-hospital syndrome are included [6, 7]. Shorter lengths of hospital stay potentially compound this as people are discharged home with on-going care needs, such as medication monitoring, wound care treatment, and limited mobility [8, 9]. This transition is particularly risky for older adults who are more likely to have multiple comorbidities and complex health and/or social care needs [10]. As such, improving the quality and safety of transitional care is a national and global priority [11, 12].

A key focus of recent research has been to identify risk factors associated with hospital readmission. Systematic reviews focusing on older patient populations identify three broad groups of risk factors - those associated with: the patient (e.g. age, male gender, ethnicity, and living conditions); the disease (e.g. morbidity, functional disability, and prior admissions); and hospital processes (e.g. length of stay, referral method, and discharge destination) [13–15].

In addition, studies have explored the quality and safety issues that arise during transitions of care, some of which are summarised by Kripalani et al [16]. They include discontinuity between secondary (acute) and primary care providers; medication errors due to different pre- and post-hospitalisation medication regimes; economic pressures whereby patients are discharge home quicker and with more responsibility for their own care; and ineffective communication between doctors and patients [16]. Delving into this, qualitative studies that specifically explore healthcare professionals’ perspectives identify fundamental system flaws that often transcend different types of transition (e.g. to home or nursing/care home), clinical populations, and organisational contexts [17–22]. Transitions of care take place within fragmented [17], under-resourced systems [17, 20, 22] where teams work to different priorities and pressures [18–20, 22]. There are few standardised systems, ways of working, or processes for delivering transitional care [18, 21, 22], and inadequate communication creates difficulties when transferring care responsibilities from one team to another [17, 19, 20, 22, 23]. Furthermore, focusing solely on medical conditions can increase risk through inadequate assessment and a lack of multidisciplinary team input [17, 18, 21, 22]. These problems are often compounded by a lack of patient and family involvement [17, 21, 23].

Numerous interventions have been developed to improve transitions of care. However, despite our understanding of the risk factors and safety issues, several systematic reviews indicate that the evidence remains equivocal as to what the most effective interventions or components may be [24–26]. Interestingly, most studies focus on what *goes wrong* at transitions in order to provide guidance and develop interventions. However, healthcare *goes right* far more often than it goes wrong [27]. Although useful, these deficit-based studies do not illuminate how staff deliver safe, high quality transitions of care or overcome the problems that they face. By learning about how safe transitions of care are managed within existing resources, we may be able to create effective intervention strategies that are both feasible and sustainable within healthcare settings.

There are only a few asset- or strength-based studies which explore how healthcare teams deliver *safe* transitional care. In the United States (US), Brewster et al. [28] identified organisational practices within high-performing hospitals that were thought to reduce readmission rates for heart failure. These included inter-disciplinary collaboration, relationships with post-acute care providers, and a culture in which staff engaged in trial and error improvement and where they perceived readmissions to be bad for patients. High and low performing hospitals did not vary in the specific, more concrete clinical practices that they used, such as follow up appointments or patient education. Also in the US, Bradley et al. [29] quantitatively identified six strategies that were associated with lower readmission rates for heart failure: partnering with community teams; partnering with hospital teams; having nurses responsible for medicine reconciliation; arranging follow up appointments prior to discharge; having communication processes in place to send discharge summaries; and assigning staff to follow up test results that arrive post-discharge. Some combinations of these strategies are represented within transition interventions and the Ideal Transitions of Care framework [30], yet it is not known which strategy/s actively contribute to reduced readmission rates [25].

This study builds on the asset-based literature to explore how high-performing general practice and hospital teams successfully deliver safe care to older adults during transitions from hospital to home. The study adopts a positive deviance approach which seeks to identify and learn from those who achieve exceptional performance on outcomes of interest [31–33]. A four staged framework has been proposed to apply positive deviance within healthcare organisations [32]. Exceptional performers are identified using routine data (stage 1) [34], and then qualitatively studied to explore how they succeed (stage 2) [35–37]. The success strategies are tested in larger more representative samples (stage 3) before being disseminated to others (stage 4). The current study addresses stage 2 of this framework. Rather than focusing on specific perspectives (e.g. hospital management [28]), specific aspects of transitional care (e.g. day of discharge [38] or communication [39]), and/or specific patient groups (e.g. heart failure [28] or stroke [39]), this study gathers multidisciplinary staff perspectives across a variety of healthcare contexts. We sought to understand what facilitates successful transitions of care within high performing teams, and the ways in which staff overcome the challenges faced in their everyday work.

## Methods

### Study design and ethics

In line with stage 2 of the positive deviance framework [32], qualitative methods were used to explore how high-performing general practices and hospital specialties succeed. Focus groups, brief observations, and interviews were conducted to explore how multidisciplinary teams support safe transitions from hospital to home for older adults. Ethical approvals were granted by the University of Leeds, UK. Full details of the methods used are available in the published protocol [40]. The study contributes to the Partners at Care Transitions (PACT) programme of research which aims to develop an intervention to improve the safety and experience of older people during transitions from hospital to home [41].

### Setting and site selection

In preparation for this study, high-performing general practices and hospital specialities that demonstrated exceptionally low or reducing readmission rates over time were identified (in line with stage 1 of the positive deviance approach). Routinely collected 30-day emergency readmission data for patients aged 75 years and over were extracted for all general practices (*n* = 151) clustered within five clinical commissioning groups (CCGs) and all cardiology, respiratory and older people’s specialties (*n* = 85) clustered within 22 acute National Health Service (NHS) Trusts in the North of England. Routine data were extracted for the most recent timeframes available (2015–17 in primary care; 2013–16 in secondary care), and binomial funnel plots were used to compare 30-day readmission rates for sites within each CCG and type of hospital specialty. High-performing sites were identified as those that exceeded the two but ideally three sigma control limits. In addition, bar charts were plotted to identify hospital specialties that demonstrated the greatest improvement (i.e. reduction) in readmission rates over time.

Up to six high-performing general practices and hospital specialties were purposively sampled to represent a range of healthcare contexts (see [40] for full details). General practices were selected using routine data regarding list size, deprivation, and the proportion of patients over 75 years / in nursing homes [42]. Hospital specialities were selected following short telephone calls with the specialty clinical leads to explore how apparent high performance may have been affected by factors associated with the data, patient case-mix, structure or resources, processes of care, and/or individual carers [43]. Where hospital specialties consisted of multiple wards, clinical leads also identified ward teams that were representative of the data (i.e. had higher proportions of over 75 yr olds and/or were perceived to perform well) and that specialty within the region (i.e. the type of treatment/care delivered).

In total, six general practices and four hospital specialties (two older people’s medicine, one respiratory, and one cardiology) participated in the study (Table 1). Data were gathered from staff who worked across 14 hospital wards including ‘base’ wards, an Elderly Admissions Unit, a ‘Delayed Transfer of Care’ ward for complex discharges, and a community hospital ward. Three hospital specialties (two respiratory and one cardiology) and two general practices did not engage with the study.

**Table 1:** Details of the high performing teams, data collection, and study participants

	High performing team	Data collected via:	Number of participants
Secondary care	Hospital A: older people’s medicine	5 x focus groups, 1 x interview, 4 x observed meetings (incorporated perspectives from 8 wards and a hospital discharge team)	32
	Hospital B: cardiology	1 x focus group, 2 x interviews, 1 x observed meeting (incorporated perspectives from 1 ward)	9
	Hospital C: older people’s medicine	3 x focus group, 3 x observed meetings (incorporated perspectives from 4 wards)	20
	Hospital D: respiratory	1 x focus group, 1 x observed meeting (incorporated perspectives from 1 ward and an integrated discharge team)	7
Primary care	General Practice A	1 x focus group, 1 2-person interview	10
	General Practice B	2 x focus groups	21
	General Practice C	3 x focus groups	20
	General Practice D	1 x focus group	7
	General Practice E	1 x focus group	7
	General Practice F	1 x focus group	5
Community care	Community trust 1	1 2-person interview (worked into/with Hospital B)	2
	Community trust 2	1 focus group (worked into/with Hospital C)	4
	Community trust 3	1 x focus group, 2 × 1- or 2-person interviews (worked into/with GP D and F)	6
	Community trust 4	5 × 1- or 2-person interviews (worked into/with GP A, B, C and E)	7
	**Total:**	**21 focus groups, 12 1- or 2-person interviews, 9 observed meetings**	**157**

In the UK, health services are broadly delivered via secondary care (acute hospitals), primary care (including general practices) and community care (including community nursing). Wider services are also available, for example, via social services and the voluntary sector. The pathways, services and infrastructure to support transitions of care vary by organisation/locality. In principle, hospital discharge is planned from the beginning of an admission. Patients are discharged from secondary care once they are deemed ‘medically fit’ (i.e. clinically optimised/stable) and ‘ready for discharge’ (i.e. necessary community support is in place). Responsibility is then handed over to primary care via discharge letters to the General Practitioner (GP). If required, care is also handed over to relevant community care or social services via referrals. Organisations can incur financial penalties for readmissions within 30-days [44].

### Participants and recruitment

Opportunity and maximum variation purposive sampling were used to recruit staff from the high-performing general practice and hospital teams (Table 1). As transitional care is not delivered by hospital and general practice teams alone, we also recruited staff from ten teams clustered within four community care trusts (organisations) that worked into and with the high-performing teams. In total, 157 participants were recruited including doctors, nurses, healthcare assistants, receptionists/administrators, allied health professionals, discharge coordinators, community matrons, district nurses, and specialist nurses. Although social care is also key to supporting transitions of care, their inclusion was beyond the scope of this clinically focused study.

### Data collection

Multidisciplinary staff focus groups lasting up to 60 min were held in each high-performing team (Table 1). The group interaction afforded by focus groups enabled multidisciplinary team members to contribute their own experiences, clarify perspectives, and to discuss issues of importance to them [45]. Where teams spanned multiple sites or included several wards, additional focus groups were conducted. Individual or two person interviews were conducted if staff were unable to attend focus groups. Suitable dates and locations for focus groups were organised via practice or ward managers. At the beginning of each focus group the researcher explained the purpose of the study. Semi-structured topic guides were used to explore shared perspectives about the concrete tools and strategies as well as the abstract cultural influences that support safe transitions of care (supplementary file 1). Focus groups and interviews were audio-recorded and transcribed verbatim, and brief field notes were written following each focus group to record contextual information such as team dynamics.

Within secondary care, brief observations of staff meetings relating to patient discharge (e.g. board rounds and multidisciplinary team (MDT) meetings) were also conducted to help researchers familiarise themselves with the ward setting and patient population, and to gather contextual information about how care transitions are planned. Observations in primary and community care were precluded by the rarity of meetings specifically relating to transitions.

Data were primarily collected by RB, a post-doctoral health services researcher, between September 2017 and May 2018. Where possible, three additional researchers with previous experience and a relevant clinical background (occupational therapist, GP registrar, and community nurse) co-facilitated focus groups or conducted individual interviews. Researchers met frequently during the data collection period to develop and discuss the topic guide and to ensure competency.

### Data analysis

A pen-portrait approach [46] was used as it facilitated large amounts of qualitative data (from focus groups, interviews, and field note) to be synthesised into rich, holistic accounts for each participating team (ward, general practice, or community team member). The four stages of a pen-portrait analysis were followed (define a focus, design a structure, populate the content, interpretation). The focus was defined as how teams successfully support transitions of care and overcome challenges. The structure included information about context, an overall summary, and detail about the key factors that were important to success. The researcher  populated the content by making notes and mind-maps to distil key information into each pen-portrait (*n* = 26). Initial pen-portraits (*n* = 5) were compared against the later ones to ensure a consistent approach, and a second researcher (RS) assessed 10 pen-portraits against the original data to ensure they provided an accurate representation. For the final stage, interpretation, RB initially generated descriptive and analytic themes for teams within primary/community care and secondary care settings and then data were analysed across settings to generate high level conceptual themes about how high-performing teams successfully support safe transitions of care. The researcher recorded analysis progression, and met with wider team to discuss emergent findings.

## Results

### Overview of findings

Staff within the high-performing teams facilitated safe transitions of care in three ways: they got to know their patients by building a holistic understanding of their care needs which was shared within and across teams; they knew each other well by building relationships within and across teams based on valuing and trusting one another; and they bridged gaps within the system to prevent things from going wrong by enhancing communication, adjusting patient expectations and adapting to competing priorities.

**Fig. 1:**
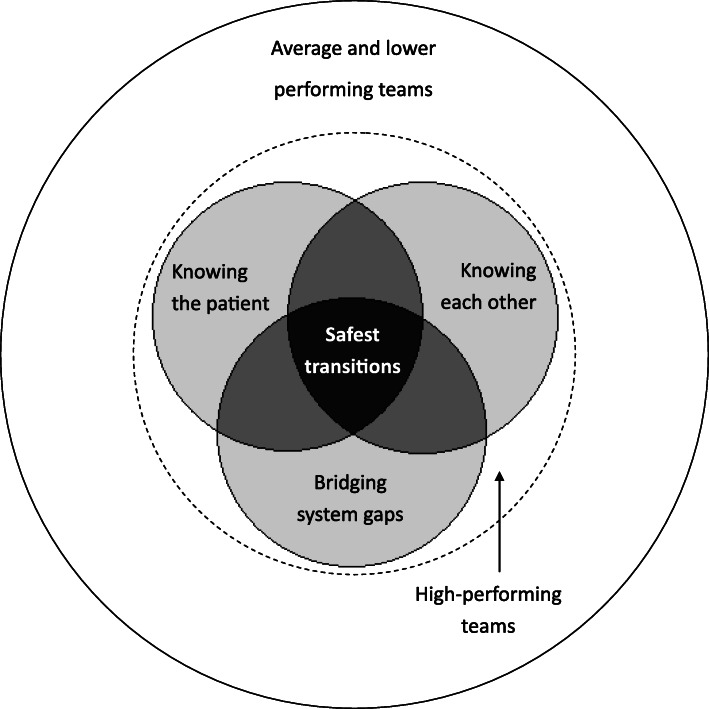
Key themes in delivering safe transitional care and the hypothesised way in which they interact

Figure 1 depicts how the three themes are hypothesised to interact to produce exceptionally safe transitions of care. The high-performing teams sit within the inner circle (dashed line). Achieving any one of these themes is hypothesised to support safer transitions of care (represented in light grey). Interactions between the themes (represented in medium grey) may further enhance safety, for example, staff felt it was easier to share knowledge about a patient’s transitional care needs if good relationships existed within teams. The safest transitions of care were thought to occur when there was evidence of all themes (represented in dark grey).

Despite this, it was challenging to achieve any one of the themes, particularly when crossing team or service boundaries. As a result, staff perceived *exceptionally* safe transitions of care to happen relatively rarely and typically only when patients had particularly complex medical/social care needs. These patients were far better known by staff who drew upon their relationships, concertedly involved a range of multidisciplinary teams, and took extra steps to bridge safety gaps. Scarce resources precluded this for every patient, consequently, at times, it is hypothesised that the high-performing teams delivered safe transitional care in a similar way to average and lower performing teams (white areas of the inner circle of Fig. 1).

Each theme is described in detail below including examples of how staff attempted to deliver safe transitional care in the most complex situations. Table 2 provides illustrative extracts from the pen portraits (the unit of analysis), and further extracts from different healthcare contexts are provided in supplementary file 2.

**Table 2:** Key themes and subthemes through which staff support safe transitions of care

Theme	Subtheme	Illustrative extracts from pen portraits
Knowing the patient	Gathering a holistic picture	**General Practice F:** Knowing the patients was particularly important to preventing readmission and was considered far more effective than using the 2% high risk and frailty registers. Knowing patients well was evident throughout the practice team but was particularly evident with receptionists. They know who is high risk/vulnerable and will often notice this and take action when patients phone (e.g. squeeze them in for appointments). Knowing patients gives them context when looking at DNA [Did Not Attend] appointments so that they can mention it to GPs and chase or investigate non-attendance (i.e. for those who are frail). ***Pen portrait Line 29***
	Building trust and rapport	**Community participants 3, 4 and 5:** Many of their nurses are able to have conversations with patients about advanced care planning decisions such as DNARs. These conversations now tend to be led by community nurses rather than GPs, and they have found that the GPs will often now rely and refer these conversations on to their nursing team. The rapport that the nurses build up with patients supports them to have these conversations. ***Pen portrait Line 75***
	A shared understanding	**Hospital A Ward B:** Timely and targeted communication is another key to success – one AHP [Allied Health Professional] described his job as a 9 h MDT meeting. Formal communication mechanisms (handovers and board rounds) enable the team to get on the same page – everyone knows what is needed, by whom, and when to support timely discharge or transfer. The team prioritise tasks and individuals are challenged but at the same time supported by the MDT to achieve the things that are required within the necessary time frame. Communication throughout the rest of the day appeared to be very integrated across the MDT. Information is cascaded to other team members / professions on the ward as required. At times this will mean one staff member communicates the same thing to multiple people (nurses, HCAs [Health Care Assistant], Doctors etc). ***Pen portrait Line 41***
Knowing each other	Feeling valued and listened to	**Community participant 2:** Based on comparisons with the other practices and her previous experience, the DN [District Nurse] Team Leader could see why the GP practice had been identified as having a low readmission rate. She perceived the general practice team to be ‘on the ball’, to work together, and to get on well. The DN felt that the GPs were approachable and that they would be listened to rather than being told ‘no’ or ‘I don’t have time’. ***Pen portrait Line14***
	Building relationships across boundaries	**Hospital B:** Each day a band 6/7 ‘ward coordinator’ (manager) works outside of the clinical staffing numbers (i.e. was supernumerary). They push things forward, liaise with others, and have time to develop relationship with the wider MDT / other services. These relationships help get things done. ***Pen portrait Line 107***
	Trusting one another	**General Practice B:** The doctors (although less so for the Practice Nurses) thought they had good relationships and trusted the DNs who are helpful, clinically good, and will escalate problems where necessary. ‘Knowing’ the DNs was key – talking to them and knowing their names. ***Pen portrait Line 94*** **General Practice F:** The palliative care nurse mentioned that when she rings the practice she is confident that things will get sorted. ***Pen portrait Line 60***
Bridging system gaps	Enhancing communication	**General Practice A:** GPs wanted to know about follow-up as soon as possible (i.e. when hospitals know a patient will be discharged) so that they could identify patients they are concerned about and plan additional care (based on their implicit knowledge about patients e.g. home circumstances). Currently they create reminders for themselves, or make appointments (e.g. on home visit list) for high risk hospitalised patients and keep putting them back if patients haven’t been yet discharged. ***Pen portrait Line 50***
	Adjusting patient expectations	**Community participant 1:** Although care would never be withdrawn from a capable patient who didn’t engage, increasingly DNs will say ‘no’ to patients, ask questions of them, set goals, signpost, and reassure patients to encourage them to self-care (rather that accepting non-engagement and doing things to patients). ***Pen portrait Line 48***
	Adapting to evolving services and competing priorities	**Hospital A Discharge Liaison team:** Multi-agency working facilitates efficient problem solving and is enabled by the teams having a better understanding of the barriers, concerns, challenges and pressures that other teams face. The teams can plan and coordinate care more effectively because they know who needs to do what, who has the specialist skills, and what everyone’s role is. ***Pen portrait Line 16***

Abbreviations: *AHP* Allied Health Professional; *DN* District Nurse; *DNA* Did not attend; *DNARs* Do Not Attempt Resuscitation; *HCA* Health Care Assistant; *GP* General Practitioner; *MDT* Multidisciplinary Team

### Knowing the patient

Staff perceived safe transitions of care to be supported by them knowing their patients well and understanding their needs. Although this was important in all teams, it differed by context. For example, general practice and delayed transfer of care ward teams felt they knew their patients ‘better’ than staff on admission or base wards.

#### Gathering a holistic picture

Staff went beyond a patient’s medical problem to holistically understand their psycho-social situation, living circumstances, and goals, worries and fears for being at home. Information was not taken at face value; staff concertedly and repeatedly dug for information from patients and families by gathering collateral and corroborating accounts. Through this staff could pre-empt and identify risks (e.g. that a spouse was struggling with care responsibilities), allowing them to make more robust discharge plans, arrange additional support, and/or deliver more effective follow-up care. Knowing the patient also enabled care to be tailored to the individual, for example, because staff would notice when patients’ behaviour was out of character.

#### Building trust and rapport

Trust and rapport was often perceived to be established by staff who had greater time to spend with patients (e.g. Health Care Assistants (HCAs), general practice receptionists, or community matrons), or by teams that provided longer-term care (e.g. complex discharge wards). It enabled patients and families to open up and be honest about their problems or concerns, which in turn allowed staff to identify support and provide reassurance. Staff observed that trust and rapport also facilitated informal conversations during which patients would mention things that they otherwise considered irrelevant to their care (e.g. information about their home environment), and the resulting relationships provided a mechanism through which patients could access the system for appropriate and timely help. In addition, building trust and rapport made it easier for staff to have difficult conversations with patients as they felt ‘safer’ broaching and being honest about issues such as prognoses or unrealistic expectations.

#### A shared understanding

Holistic patient knowledge was shared among team members so that others could pick up care as needed. Ward teams had a shared awareness of discharge plans, while general practice and community teams knew which patients were likely to be vulnerable following discharge. This knowledge was distributed across the multidisciplinary team including unqualified staff and those who were not regular team members. Regardless of the setting, awareness was facilitated in similar ways; formal meetings helped teams create transitional care plans, while daily board rounds, huddles, and/or handovers enabled staff to update one another, raise pertinent issues, and prioritise tasks. If staff understood how a patient’s care had progressed, and/or had been involved in decisions, teams could take calculated risks at transitions. Without this shared knowledge, staff were perceived to be more reactive and risk averse. For example, Out of Hours staff would readily re/admit patients to hospital, or hospital staff covering weekends would take less responsibility for discharge decisions. Informal communication was considered key to sharing nuanced information. However, there were limited mechanisms to facilitate this, and so in depth patient knowledge was often held within rather than across team boundaries.

### Knowing each other

Safe transitions of care were perceived to depend not only on knowing the patient well, but also on knowing other team members and those from different teams and settings.

#### Feeling valued and listened to

Within teams, positive relationships were thought to develop when staff felt valued and listened to. Relationships across multidisciplinary groups were facilitated by integrated and non-hierarchical ways of working; staff actively involved each other in open discussions and they sought and respected the professional perspectives and experiences of others. This was considered particularly important when staff worked remotely (e.g. community nurses) or on the periphery of teams (e.g. therapists). When staff felt valued and listened to they approached one another to ask questions and seek advice, and they felt comfortable raising concerns and/or challenging team members. Everyone contributed their own ‘piece of the jigsaw’ to create robust transitional care plans.

#### Building relationships across boundaries

Although relationships less commonly existed across teams and service boundaries, similar benefits to those described above were observed - staff could approach others, share perspectives, raise concerns, and seek advice. Certain staff (e.g. discharge coordinators, senior nurses, therapists, and community matrons) proactively used the scope of their roles to build relationships across teams and settings. Relationships were bolstered when staff were receptive and responsive to each other, i.e. by helping and supporting colleagues or responding to requests promptly. When relationships existed across boundaries, information flowed more easily via informal routes and staff felt valued, particularly if they came from different professional backgrounds.

Both within and across teams, relationships seemed to be facilitated by proximity. Regular meetings that brought staff from different teams together helped develop and maintain relationships. Meeting face to face provided opportunities to interact socially as well as professionally which in turn promoted informal communication to support safer transitions. Although staff couldn’t always attend these meetings due to work load or location, others would try and attend in their place, or staff would proactively update each other afterwards. Knowing each other also provided a means of influence. Staff found it easier to communicate verbally with people that they knew and this was considered more persuasive and effective than written or electronic communication. Staff drew upon relationships across boundaries to elicit the action they required, particularly when they were concerned about a patient, or when prompt action was needed.

#### Trusting one another

Trust was thought to underpin many of the relationships within and across teams. It was important that staff trusted each other’s judgements and took ownership of actions. Within teams, this was best exemplified by discharge coordinators whose role was considered most effective when staff, particularly senior nurses, trusted them to navigate the discharge processes, their team’s ways of working, and to work autonomously. Trust was disrupted when discharge coordinators rotated between wards and, as a consequence, nursing staff would double check their actions or simply do things themselves to ensure safe transitions. Although trust was more difficult to gain when people worked across services, it facilitated more collaborative and coordinated patient care, for example, if community therapists trusted hospital assessments and referrals.

Relationships were difficult to maintain in a context of increasingly stretched services and workloads, and organisational structures and processes exacerbated these problems. Disruption to teams (e.g. over weekends), regular staff rotations, and the reorganisation of services (e.g. moving community teams out of general practices) were detrimental. At times, relationships were maintained by team leaders who proactively built links and/or took temporary preventative action to ensure safe care during periods of destabilisation.

### Bridging gaps in the system

Across all settings, staff attempted to bridge gaps within the system to improve transitions of care. They did this by trying to enhance communication, adjust patient expectations, and by adapting to evolving services and competing priorities.

#### Enhancing communication

General practice and community staff often reported receiving late, inaccurate, and/or unclear discharge letters and referrals. Several hospital ward teams tried to bridge this gap by improving the quality of their discharge letters or sending additional letters to communicate plans and decisions in more detail. Referrals were written close to discharge to ensure they contained accurate information, and staff ensured services were in place before discharging patients home. General practice and community staff felt that they relentlessly chased and clarified poorly communicated information either directly from the hospital teams or by piecing together information from patients, families and/or other primary care teams (e.g. pharmacies). Some GPs reviewed the discharge letters of patients they had seen most recently, and certain teams created their own tracking systems to identify discharges in a timely manner.

Although various service level interventions (e.g. electronic discharge letters/care records and centralised referral hubs) helped communicate critical information, they were not always considered a sufficient alternative to verbal communication that was facilitated through relationships, particularly when care was complex. In these situations staff often combined electronic communication with a telephone call to verbally hand patients over. Furthermore, staff improved continuity and communication by actively involving patients and families. Hospital staff provided information and signposted community support. Community and general practice staff actively encouraged and, at times, relied on patients and family to inform them of discharge, provide information (e.g. discharge letters and/or medication boxes), and raise concerns.

#### Adjusting patient expectations

At times, staff felt that patients and/or families had unrealistic expectations about their care. This disparity was perceived to be exacerbated by service pressures and constraints which minimised the amount of time staff could spend with patients and/or created referral delays. In addition, patients were sometimes actively prevented from having a role or self-managing their care by the way in which nurses are ‘trained’ to do things *for* patients and by some policies and guidelines (e.g. administering certain medications). To overcome this and maintain safety, staff tried to adjust patient and family expectations by challenging perceptions about patient and staff roles. Most hospital wards tried to encourage an active patient role (e.g. through End PJ Paralysis campaigns) although found this challenging, particularly when people were older or acutely unwell. The ‘little things’ were perceived to work best - like asking patients to pour water for themselves, or by subtly changing language to encourage patients to get up and dressed. Longer stay wards trained patients and families in aspects of their care, such as medications and feeds.

Staff also addressed inappropriate service use. If transitions were particularly complex, or if there was a large disparity between staff and patient/family expectations, hospital staff would hold meetings or proactively ask the multidisciplinary team to reinforce key messages over time. In primary and community care, staff educated patients and families following discharge and proactively monitored and supported individuals by scheduling regular appointments or allocating key workers. Care plans provided a ‘contract’ as to how patients should manage and maintain their own health. At times, community and general practice staff felt that unrealistic expectations were exacerbated by hospital teams over promising or not explaining things to patients (e.g. that the community nurse could not help wash and dress them as the hospital nurses had done). These problems were minimised – but not eradicated – when staff across settings understood one another’s roles.

#### Adapting to evolving services and competing priorities

Finally, staff attempted to keep up-to-date with regular changes to health, social, and voluntary services through informal conversations. These were most effective in stable and experienced teams and/or when relationships existed across settings. General practice teams often had staff (e.g. practice managers) who would intentionally identify and disseminate changes or actively invite local services in to talk to them.

Safety was also thought to be jeopardised by competing priorities that existed within (e.g. balancing patient flow in hospital) and across services (e.g. working to different goals, processes and policies). Although it was often difficult to overcome competing priorities, staff engaged with teams more appropriately and mitigated problems if they understood one another’s roles – what other teams could offer and the pressures and constraints that they faced. At times, staff pushed back against hierarchies or power, they engaged service leaders to identify solutions, and some community teams up-skilled their nurses (e.g. in prescribing medicines) to minimise their reliance on other teams.

Across settings staff had different perspectives on the problems that they faced. Hospital teams predominantly perceived avoidable readmissions to result from primary and/or community care failings, whereas primary and community care staff felt that hospitals lacked responsibility for patients post-discharge. There were few, if any, opportunities to share concerns, feedback and/or facilitate learning across settings and staff felt powerless to affect change. When staff did take action to bridge these gaps, the solutions tended to be situation specific rather than systemic, and so efforts were generally only made for particularly complex transitions of care.

## Discussion

Three key themes were thought to facilitate safe transitions of care: staff got to know their patients well; they capitalised on and sought to develop relationships with one another and; they attempted to bridge system gaps. Staff faced various challenges in achieving these three themes, some of which they could only attempt to overcome when caring for patients with particularly complex needs.

### Building relationships with patients and families

Involving patients and families in care is a central tenet of healthcare policy globally [47, 48] and may be key to enhancing safety at transitions [26, 49]. However, the extant literature predominantly focuses on the need to *provide patients and families* with information and to communicate discharge plans [17, 23]. This is undoubtedly important, but this study also emphasises the key role that patients and families can play in *providing staff* with information - and the conditions under which this is facilitated i.e. when relationships are established.

Familiarity with the patient has previously been shown to impact the quality of knowledge sharing, assessments, and decision making within teams at transitions [38, 50]. Furthermore, Waring and colleagues have found that safer, more patient-centred discharges from stroke and hip fracture wards are facilitated by incorporating holistic patient knowledge (psychological, social and personal needs) with traditional biomedical knowledge. Importantly, this holistic knowledge was gathered through informal interactions with patients and families [51], highlighting the importance of building relationships with patients. This finding is supported by Simms-Gould et al. [52] who found staff use investigative skills to gather information and collateral accounts to support safe transitions.

Providing staff with information may be one way in which patients and families can support and scaffold the quality and safety of their care [53, 54]. Without overburdening them, initiatives could effectively support patients and families to complete this ‘work’, for example, by educating them on the types of information that healthcare professionals value and/or need to know. Supporting patients to do this will be essential as they do not always feel comfortable or skilled in relaying information, especially when unwell and/or in busy hospital environments [55]. Structural changes, for example re-configuring staff rotas may also help enhance continuity and facilitate the development of trust and rapport between patients and staff.

### Building relationships with staff

The importance of working within inter-professional, integrated, non-hierarchical teams is well documented, both in general and in relation to transitions of care (e.g. [35, 51, 56]). This study suggests that integrated working may be underpinned by the relationships and trust that staff build within and across teams, and that this is often facilitated by proximity and informal interactions. Previous research suggests that staff build relationships across settings specifically to overcome uncertainty and discontinuity at transitions [52]. Staff also perceive relationships and communication to improve as a result of physically bringing people together for meetings [17]. However, in addition to formal meetings, informal interactions may enable staff to share experiential knowledge, develop mutual understanding, and build trust to enhance collaboration at discharge [51].

Technology is often proposed as a way of enhancing safety at transitions, for example, by developing electronic patient records to facilitate communication and coordination between staff/teams who work remotely [22]. Technological solutions are undoubtedly required but they do not necessarily provide a ‘magic bullet’. In fact, electronic patient records have previously been found to unintentionally, negatively impact interpersonal communication by facilitating discharge through computers rather than encouraging the interaction of multidisciplinary team members [18]. Further to this, healthcare policy in the UK and further afield emphasises the need to develop integrated care systems (e.g. the NHS Long Term Plan [57]) and interventions to improve care coordination. However, achieving integration has been slow [58] and interventions rarely demonstrate unequivocal positive effects [24]. In part, this may be due to fundamental cultural differences between healthcare setting [58], but robust evaluations are required to understand *how* interventions work in some contexts but not others.

Findings from this study highlight that safe transitions of care may depend on informal communication facilitated through staff relationships. Contextual and cultural factors (such as relationships) are therefore likely to be important [28] and, as such, priority should be given to help facilitate conditions under which staff relationships and collaboration can flourish [56]. System changes could better support staff to build and maintain relationships with one another, for example, by minimising staff rotations across hospital wards and the relocation/reorganisation of primary and community care teams [59]. Alternatively, organisations may be able to improve relationships and collaboration within their own workforce by recruiting people based on their values and beliefs.

### Bridging gaps in the system

In this study, the resilient actions of healthcare teams (e.g. verbally communicating risk or chasing missing information) helped bridge gaps in the transitional care system. Informal, verbal handovers are known to overcome formal communication systems deficiencies, but it is also recognised that staff cannot do this for all patients [39, 52]. Although these bridging actions improve safety, they only provide a temporary fix typically for specific individuals with more complex needs. In fact, it could be argued that these bridging actions shift resources (e.g. staff time) away from other patients within the system. To enhance safety for a wider range of patients, not just those with particularly complex needs, structural and systemic changes are required to overcome common system inadequacies.

Understanding peoples’ roles and constraints helped staff mitigate transitional care problems by allowing them to engage appropriately with other teams and adjust patient expectations. However, staff often struggled to achieve this. Staff don’t always understand how care is delivered in other parts of the system and how their actions positively or negatively impact this [52, 60]. Bringing people together from different settings may help improve transitional care. For example, inter-professional simulation has helped staff recognise different professional perspectives and appreciate the challenges faced when transitioning older people from hospital to home [56]. Through this staff reported improvements in communication across settings, use of available services, and behavioural changes such as earlier discharge planning and taking a more holistic approach [56].

Staff also reported having few opportunities to learn across settings, and limited agency to affect change. The ability to learn is key to a positive patient safety culture [61–63]. Furthermore, high-performing US hospitals that successfully reduce readmission rates have engaged in trial and error learning and built relationships with post-acute providers in order to share expertise and data [28]. As such, NHS trusts and CCGs may have an important role in highlighting exceptional performance and supporting a learning environment. Healthcare organisations could provide opportunities for shadowing or job swaps to give staff experience of delivering care in a different setting. However, this would need to be balanced against the potentially negative impact of disrupting teams. The findings also suggest a need to embed accessible and visible mechanisms that support learning across settings so that frontline staff feel empowered to influence care and generate change. By better understanding the pressures, constraints and competing priorities that other people face, staff may be able to systematically bridge system gaps that make their jobs more challenging.

### Study limitations

It is important to reflect on the study’s limitations. First, the teams may not have truly demonstrated exceptionally high performance as the validity of emergency readmission data can be questioned [64, 65] and case-mix/service level differences could not be fully adjusted for. Moreover, the high performing hospital and general practice teams were not linked to one another, which may have contributed to contrasting or contradictory views about how safe care was delivered.

Second, in terms of the trustworthiness, the credibility of findings (i.e. confidence in their ‘truth’) can be questioned [66]. People become habituated to their everyday work when things go right far more frequently than they go wrong [27]. As such, participants may not have been able to articulate what they do to succeed. Staff may also have revealed what they are ‘meant’ to do (aligned to policies and procedures etc.) rather than what they actually do, and/or some may have felt inhibited during discussions due to the focus group method [45]. Although data were, to some degree, gathered from multiple sources and triangulated (via focus groups, interviews, and observations), prolonged engagement with sites via ethnographic methods may have helped overcome these challenges [66, 67]. In addition, data saturation may not have been reached despite the large amount of data collected [68].

Third, the findings may have lacked transferability (i.e. applicability to other contexts) [66]. Although purposive sampling was used and findings were member checked with key stakeholders, some high performing teams did not engage with the study and participating staff may not have represented their multidisciplinary teams. Furthermore, teams from settings/specialties that were not included in this study may deliver safe transitions in different ways.

Fourth, the findings may have lacked confirmability (i.e. then extent to which they could be corroborated). The researchers will have held their own biases and motivations which will have been influenced by the overarching aims of the wider programme of work [66]. Furthermore, a pen portrait analysis was conducted to generate high level conceptual themes, however, analysing the source data may have led to more descriptive findings. Our use of pen portraits represents an adaptation of how the analytic technique was used in its original incarnation. Pen portraits allow the researcher to integrate multiple sources of qualitative data and so are often used in longitudinal qualitative analyses [46]. The authors of the technique were consulted prior to its use in a static analysis and offered advice on how to pursue this. As such, we did not encounter any specific challenges related to this. Our broad research question did, at times, make it challenging to define a structure and populate the pen portrait content. The method though explicitly advises against creating rigid structures [46], and we rigorously second checked the pen portraits to ensure they accurately represented the raw data.

## Conclusions

Staff perceived that safe transitions were underpinned by three key themes: knowing the patient, knowing each other, and bridging gaps within the system. Cumulatively these themes were hypothesised to interact to produce exceptionally safe transitions of care, although staff faced extensive challenges in achieving them on a routine basis. Staff were typically only able to overcome these challenges when caring for patients with particularly complex needs. Thus, the safest transitions of care were often perceived to be delivered to those patients that were most vulnerable. By engendering system changes that support staff to get to know their patients, know each other, and bridge gaps more frequently, healthcare organisations may support staff in delivering safer transitions of care to a much wider range of patients.

## Supplementary information

**Additional file 1.** Discussion guides.

**Additional file 2.** Illustrative extracts from pen portraits to exemplify each factor within different healthcare contexts.

## Data Availability

The datasets used and/or analysed during the current study are available from the corresponding author on reasonable request.

## Abbreviations

AHP: Allied health professional
CCGs: Clinical commissioning groups
DN: District nurse
DNA: Did not attend
DNARs: Do not attempt resuscitation
GP: General practitioner
HCA: Health care assistant
MDT: Multidisciplinary team
NHS: National health service
UK: United Kingdom
US: United States
