# Supplementary material for: Delivering exceptionally safe transitions of care to older people: a qualitative study of multidisciplinary staff perspectives
Source: BMC Health Serv Res. 2020 Aug 24;20:780. doi: 10.1186/s12913-020-05641-4 (PMC7444052; doi:10.1186/s12913-020-05641-4)
Supplement: Supplementary file 2 — Additional file 2. Illustrative extracts from pen portraits to exemplify each factor within different healthcare contexts. [file 12913_2020_5641_MOESM2_ESM.doc]

**Supplement 2: Illustrative extracts from pen portraits to exemplify each factor within different healthcare contexts**

| **Theme** | **Subtheme** | **Illustrative extracts** |
| --- | --- | --- |
| Knowing the patient | Gathering a holistic picture | **General Practice F:** Knowing the patients was particularly important to preventing readmission and was considered far more effective than the 2% high risk and frailty registers. Knowing patients well was evident throughout the practice team but was particularly evident with receptionists. They know who is high risk/vulnerable, will often notice this and take action when patients phone (e.g. squeeze them in for appointments). Knowing patients gives them context when looking at DNA appointments so that they can mention it to GPs and chase or investigate non-attendance (ie for those who are frail). ***Pen Portrait Line 29***  **Hospital C Ward B:** Having a holistic picture is key to safe transitions. Staff assess past, present and future situations taking into account the wider circumstances not just the reason for an admission. They do this in several ways. Staff can access community notes to see how patients have been discharged in the past. OTs look for ‘red flags’ and dig deep to identify things that don’t stand out as an immediate concern. They follow up the initial work that previous teams have done and often uncover a much bigger picture. Other wards don’t have time to deal with the sorts of problems that they uncover. They continually look for collateral information from various sources. ***Pen Portrait Line 90***  **Hospital A Ward A:** Truly knowing the patients and having relationships with them was also considered key to facilitating a successful discharge. It was important to listen to the patient so that staff could understand their aims and wishes and find out about any worries or fears that they have for discharge. ***Pen portrait Line 37***  **Community participants 3, 4 and 5:** Compared to the traditional, task orientated DN role, their visits are much more holistic. They make multiple assessments about a patient’s holistic situation and often find that one assessment will lead into another etc. They will always put care plans in place following an assessment and, even when something looks like a simple referral, they will often uncover more by digging a bit deeper. ***Pen Portrait Line 55***  **Community participant 12:** Regardless of the reason for referral, all new patients are given a 1.5 hour holistic assessment which assesses all aspects of the patients health and social care. This is very time consuming, however, it may help them identify and deal with other problems that they need to address and that haven’t been picked up by the hospital teams. Rather than being task orientated (e.g. simply deal with pressure care) this holistic assessment may prevent readmissions. ***Pen portrait Line 66*** |
|  | Building trust and rapport | **General Practice B:** Particularly when many clinicians are involved in their care, patients are confused about who to contact but their relationships make the practice a first point of contact (which can mean the team are working at their limit). Patients often disclose problems during routine appointments and staff identify problems / high risk patients when they notice things that are out of character for an individual. ***Pen Portrait Line 79***  **General Practice D:** Familiarity and knowing patients well supports post discharge care. They are a small practice with a steady population. Reception staff often get more involved (patients tell them more than they usually would), and as they have a good background knowledge of a patient they can identify important things and have greater awareness of the urgency. They escalate to the doctors (same day) when needed. Reception also often have long conversations with patients (who are often isolated) which means a lot to the patient and helps reception get to know them. ***Pen Portrait Line 51***  **Hospital A Ward A:** Sometimes staff have to dig for the information that is needed (e.g. because patients or families don’t realise it is important information) and at other times it is necessary for staff to build trust with the patients/families in order to uncover information and better understand the home situation. Therapists seemed to be in the best position to do this as they spend longer with the patient and have more opportunity to discuss things with them / build trust – e.g. when patients have rests between exercises. ***Pen Portrait Line 44***  **Hospital C Ward B:** On discharge, patients are told to contact their GP or, if they have med queries, the ward. Patients will often contact the OTs, sometimes months after discharge, for good and bad reasons. This is often the result of the relationships that they have built. ***Pen Portrait Line 60***  **Community participants 10 and 11:** they are able to spend a long time with patients, talking to them, and getting to know their circumstances and support networks. This enables patients to get to know them, open up more effectively and express their fears and concerns. ***Pen Portrait Line 57***  **Community participants 3, 4 and 5:** Many of their nurses are also able to have advanced conversations with patients about decisions such as DNARs. These conversations now tend to be nurse rather than GP led, and they have found that the GPs will often now rely and refer these conversations on to the nursing team. The rapport that the nurses build up with patients supports them to have these conversations. ***Pen Portrait Line 75*** |
|  | A shared understanding | **General Practice C:** They generally identify high risk patients through group discussion at a weekly team meeting and via a nurse weekly team meeting. Staff bring patients to discuss so that the whole team is aware of the patients and their situations. This means that anyone can care for a patient as they have an idea of their background already. ***Pen Portrait Line 48***  **General Practice D:** Even if patients have no active needs, they are placed on the GSF so that awareness is raised among DNs, Palliative care nurses, and CMs. Through this meeting, and during team discussions, they try to identify patients that staff have concerns about. ***Pen Portrait Line 46***  **Hospital A Ward B:** Timely and targeted communication is another key to success – one AHP described his job as a 9 hour MDT. Formal communication mechanisms (handovers and board rounds) enable the team to get on the same page – everyone knows what is needed, by whom and when to support timely discharge or transfer. The team prioritise tasks and individuals are challenged but at the same time supported by the MDT to achieve the things that are required within the necessary time frame. Communication throughout the rest of the day appeared to be very integrated across the MDT. Information is cascaded to other team members / professions on the ward as required. At times this will mean one staff member communicates the same thing to multiple people (nurses, HCAs, Drs etc). ***Pen Portrait Line 41***  **Community participants 3, 4 and 5:** Their team is very integrated and includes DNs/nurses, physios, OTs, CMs, PCNs and social workers. They are all based in the same building which enables everyone to communicate and discuss things more effectively. Their team have a daily handover where they can discuss patients, raise queries/questions and make decisions on how best to escalate care. Also, being in the same building has reduced referral times, as the discussions enable everyone to get on the same page about a patient quite quickly. ***Pen Portrait Line 79***  **Community participants 6 and 7:** The DNs considered themselves to be the key reason for patients not being readmitted (along with other community teams e.g. palliative care). They act as a liaison for additional care needs e.g. by alerting GPs to problems, liaising with care agencies, and trying to get the right people/agencies involved. ***Pen Portrait Line 43*** |
| Knowing each other | Feeling valued and listened to | **General Practice C:** The ability for staff to identify and raise concerns appears to be facilitated by the team’s culture. The practice has a very low turnover and network with each other across the two sites. There is no hierarchy so staff approach each other and ask questions regardless of role. The team feel that they can rely on each other if they have a problem. ***Pen Portrait Line 79***  **Hospital C Ward C:** The team spend a lot of time gathering information. They valued a strong MDT approach as everyone asks different but necessary questions that feed into good discharge plans. […] Each discipline contributes valued specialist input, then they come together, ‘working as one’ to deliver joint treatment and joint discharge planning. ***Pen Portrait Line 115***  **Community participant 2:** Based on comparisons with the other practices and her previous experience, the DN Team Leader could see why the GP practice had been identified as having a low readmission rate. She perceived the general practice team to be ‘on the ball’, to work together, and to get on well. The DN felt that the GPs were approachable and that they would be listened to (not told ‘no’ or ‘I don’t have time’). ***Pen Portrait Line 14***  **Community participant 12:** Their community team has a monthly case management meeting with the full MDT including social services, clinical care coordinators, 3^rd^ sector, and a community geriatrician. This provides an opportunity to ‘get heads together’ and hear other people’s views, opinions and ideas. […] As the community geriatrician attends their monthly meeting they have an opportunity to get to know them and spend time face to face which enables them to have discussions more easily. ***Pen Portrait Line 44*** |
|  | Building relationships across boundaries | **General Practice B:** Links with other services were also important. […] The DNs currently have good leadership but years of reorganisations have reduced face to face contact and broken down relationships. The team have tried to maintain these relationships as best they can. ***Pen Portrait Line 94***  **General Practice C:** The community matron felt that the general practice team was very inclusive and welcoming and that she was part of the MDT rather than an outsider. ***Pen Portrait Line 32***  **Hospital B:** Each day a band 6/7 ‘ward coordinator’ (manager) works outside of the numbers. They push things forward, liaise with others, and have time to develop relationship with the wider MDT / other services. These relationships help to getting things done. ***Pen Portrait Line 107***  **Hospital A Ward C:** The DLOs know what information everyone that is involved in a discharge needs and they know who to ask in order to get it. It works best when the coordinating staff member is embedded within the team as they get to know how everyone works etc. ***Pen Portrait Line 52***  **Community participant 12:** Relationships help facilitate much of what they do within their roles. Good relationships with general practice teams enable them to initiate management plans or treatment and facilitate ‘good open discussions’ about patients. Relationships are built by GPs being approachable and available to speak to them or getting back to them when needed. ***Pen Portrait Line 17***  **Community participant 1:** The CM tries to build relationships with the GP practices that they cover by popping in, but this is a challenge on their time as they cover several practices. ***Pen Portrait Line 75*** |
|  | Trusting one another | **General Practice B:** if [staff] identify a potential problem, they take ownership and deal with it then rather than leave it to become a bigger problem (e.g. for the nurses) later on. Ownership of patients was key ***Pen Portrait Line 66***  **General Practice F:** The palliative care nurse mentioned that when she rang the practice she was confident that things would get sorted. ***Pen Portrait Line 60***  **Hospital A Discharge Liaison team:** The team perceived multiagency working to be strongly underpinned by relationships and trust. The team work daily with social workers and district nursing (for EoL care) and are continuing to build networks and trust with staff from the other teams. This enables the team to have challenging conversations (with staff and families) and facilitates the coordination of care […] They perceived a lot of the multiagency working, relationships and trust to be facilitated by face to face working e.g. through meetings. ***Pen Portrait Line 22***  **Hospital A Ward B:** Staff perceived trust and team stability to be central to their success. As most (senior) staff have been in the team for a long time, staff know each other really well, they know who to go to, and know who does what within the team. Entwined with this is trust – the team trust (and respect) one another so that when staff raise concerns they are listened to, staff know that people will do what they say etc. ***Pen Portrait Line****22*  **Community participants 8 and 9:** When the SN or CM feel it is not possible to wait for equipment they will email the deputy nurse who will advise them how best to bypass the panel. They did not think that this workaround was common knowledge and thought that it only happened because the deputy nurse knows them and trusts that they need what they say they need – ‘it’s not what you know, it’s who you know’. ***Pen Portrait Line 117*** |
| Bridging system gaps | Enhancing communication | **General Practice A:** GPs wanted to know about follow up as soon as possible (i.e. when hospitals know a patient will be discharged) so that they could identify patients they are concerned about and plan additional care (based on their implicit knowledge about patients e.g. home circumstances). Currently they create reminders for themselves, or make appointments (e.g. on home visit list) for high risk hospitalised patients and keep putting them back if patients aren’t yet discharged. ***Pen Portrait Line 50***  **General Practice E:** Hospitals facilitate continuity by making direct referrals (e.g. community matrons) and by phoning GPs to hand over complex patients – to explain what has changed / happened and to raises awareness of patient needs so that better support can be provided. ***Pen Portrait Line 29***  **Hospital C Ward A:** No-one trusted paper referrals reaching the intended person / being acted upon, or thought that written communication was effective enough for complex patients. Written information was easy to ignore. The doctor would sometimes call GPs with important information rather than relying on a line in the discharge letter. […..] Verbal communication conveys information more effectively, entices people into the problem, and was considered more difficult to ignore (therefore you get the answer you are looking for and get things done). ***Pen Portrait Line 62***  **Hospital B:** Verbal handovers and referrals for complex patients are important as there is only so much that can be written down (eg when referring to District Nursing or community rehab). Verbal handover enables ward staff to ensure onward services are aware of patient risk. Community sometimes phone the ward to clarify information or discharge error, or a District Nurse who has been seeing the patient for a long time may call the ward to update them. Ward staff struggle to speak to specific DNs as the service is centralised. ***Pen Portrait Line 40***  **Community participant 13:** The DN often has to phone the ward for more information which is extremely time consuming as the nurse who wrote the referral is not always on shift, notes are not available, and ward staff have to ring them back. ***Pen Portrait Line 40***  **Community participants 8 and 9:** They often communicate with GPs and within the community teams via tasks (which are sometimes followed up by a verbal conversation). Tasks make communication easier as staff don’t have to ring or wait outside of surgeries, they provide a trail of communication, and they ensure that people know what they are doing. The very good practices respond quickly to tasks or phone calls from the community staff and, although all practices have a ‘bypass telephone line’, these practices will actually answer it. Hospital wards rarely communicate concerns or information about complex patients to their team verbally (e.g. when they are about to be discharged). Although there are many benefits to electronic communication, they felt that it can ‘lose something’ compared with communicating with someone verbally. ***Pen Portrait Line 57*** |
|  | Adjusting patient expectations | **General Practice C:** The practice conducts a quarterly audit of readmissions and frequent flyers and will proactively contact patients who appear to use services inappropriately. Where this is believed to be the case, they will try to educate patients on who to contact within the practice for support and / or will designate a lead GP to build continuity and give the patient confidence in the GP and practices ability to help them. ***Pen Portrait Line55***  **General Practice D:** Hospital teams promise things that can’t be delivered which causes stress to patients and results in practice staff being blamed. Hospitals possibly misunderstand what is possible within community services. ***Pen Portrait Line 20***  **Hospital D Ward A:** EDD is also shared with patients (e.g. via bedside boards) so that patients and families know what they are working towards and can prepare for discharge. This helps set expectations but can cause problems when dates change (e.g. move forward). ***Pen Portrait Line 37***  **Community participants 6 and 7:** families can also be unrealistic about how hard it is to care for someone 24hrs a day and so they do a lot to identify carer strain and signpost support for it. The DNs try to frame care packages as a supportive mechanism rather than indication of family failure, they reiterate and continually revisit the support that is available to people as situations change and carers become more strained, and they will emphasise the importance of carers looking after their own wellbeing in order to better support the patient. ***Pen Portrait Line 55***  **Community participant 1:** Although care would never be withdraw from a capable patient who didn’t engage, increasingly DNs will say ‘no’ to patients, ask questions of them, set goals, signpost, and reassure patients to encourage them to self-care (rather that accepting non-engagement and doing-to). ***Pen Portrait Line 48***  **Community participant 13:** Sometimes, though, individual trust policies prevent DNs from being able to promote self-care with patients, for example, policies relating to the use of certain drugs in home settings. ***Pen Portrait Line 63*** |
|  | Adapting to evolving services and competing priorities | **General Practice E:** Secondary care lack an understanding primary care. Despite extensive discharge planning, hospitals are naïve to patients realistic discharge needs. It is much more complex than simply ensuring that everything is in place […] They thought hospitals perceived general practice to be a ‘dumping ground’ and that this means things are missed. Hospitals should take equal responsibility for arranging onward care / treatment e.g. referrals to podiatry, pharmacy etc. They weren’t sure whether this was a lack of understanding about GP capacity or a lack of access to do these things themselves. Doctors rotating into primary care was perceived to help reduce misunderstandings. ***Pen Portrait Line 43***  **General Practice C:** Services change very rapidly but they considered their practice to be relatively ‘switched on’ about the available support, mainly as a result of their PM who emails information to the team. In addition, they arrange for services to come in and talk to their patient group with the intention that they will then spread the information to others in the community. ***Pen Portrait Line 127***  **Hospital A Discharge Liaison team:** Multiagency working facilitates efficient problem solving and is enabled by the teams having a better understanding of the barriers, concerns, challenges and pressures that other teams face. The teams can plan and coordinate care more effectively because they know who needs to do what, who has the specialist skills, and what everyone’s role is. ***Pen Portrait Line 16***  **Hospital A Ward B:** At times, safe discharge and top down pressure to maintain flow conflict resulting in inappropriate ward transfers prior to discharge. They will stop discharges if necessary (e.g. late at night) but sometimes have no control over this. ***Pen Portrait Line 16***  **Community participants 10 and 11:** The PCNs experience of previously being DN team leaders gives them a good understanding of the DN role. Practices that better understand their roles tend to be more engaged with their service. They invite the PCNs to regular GSF meetings (GPB and C), and through these they are able to have two way conversations. The more engaged GPs will respond to their requests and work with them to care for patients (GP A, B and C). ***Pen Portrait Line 41***  **Community participants 13:** The DNs do a lot to support and promote self-care with patients. The DN felt that families can provide a support network and are able to fill in the gaps between health and social care provision. Sometimes, though, individual trust policies prevent DNs from being able to promote self-care with patients, for example, policies relating to the use of certain drugs in home settings. ***Pen Portrait Line 60*** |
