# Supplementary material for: Delivering exceptionally safe transitions of care to older people: a qualitative study of multidisciplinary staff perspectives
Source: BMC Health Serv Res. 2020 Aug 24;20:780. doi: 10.1186/s12913-020-05641-4 (PMC7444052; doi:10.1186/s12913-020-05641-4)
Supplement: Supplementary file 2 [file 12913_2020_5641_MOESM1_ESM.doc]

Supplementary file 1: Discussion guides

Partners At Care Transitions (PACT): Understanding excellence from a secondary care perspective.

Focus group / interview framework

Things to consider:

- Transitions from hospital to home rather than care home
- The full multidisciplinary team including AHPs, pharmacists, external teams etc.
- We are trying to understand not only *what* you do, but also *how* that helps your team. We will constantly be asking you 'why'!
- Do you do anything differently to other similar wards?

What do you think a really good discharge looks like [for an older person]? (flipchart)

On your ward, which of these things do you think you do well? – highlight on the flipchart

---

Take each 'thing' in turn:

Practicalities:

- What actually happens in xxx / what does xxx look like / how do you do xxx
- Is this any different to other wards?

Function:

- What is the purpose of xxx / What does it do xxx / Why do you do it like that?
- Sometimes easier to look at the negative – what would be the consequence of not doing xxx?

What pressures and challenges do you face and how do you overcome them?

From the 'quick fire list'

- Are there any gaps and why? During admission, on discharge, post discharge?
- What are the challenges and how do they try to overcome them?
- Links with other teams?

Nothing ever goes exactly to plan! What can throw a spanner in the works and how do you cope with that?

How do patients and their families contribute to a good discharge?

How do you encourage or support patients and their families to contribute?

Partners At Care Transitions (PACT): Understanding excellence from a general practice perspective

Focus group / interview framework

Things to consider:

- Transitions from hospital to home rather than care home
- Consider the full multidisciplinary team including nurses / community / external teams
- We are trying to understand not only *what* you do, but also *how* that helps your team.

So far I've referred to patients who move from hospital to home as going through a 'transition of care' but everyone has different perspectives and uses different terminology. **What do you consider a transition of care to be?**

Everything is a bit easier when you can tell a story! Can you think of an older person that you have cared for who has frequently been in and out of hospital? If you can think of a patient that you all know then that is great, but if not your own example is absolutely fine. Briefly describe the situation to everyone. **How was this patient supported during that time?**

Can you think of a similar patient that you have successfully and appropriately managed to keep at home? **What was different about this situation and how did that help you?**

So were now going to discuss transitions more generally. **How do you identify older people who are at risk of being admitted or readmitted to hospital?**

Once an elderly patient has been discharged from hospital, how do you monitor or keep track of their health or progress?

In an ideal world, what sort of things would you like to know and when? How would this help?

I want you now to try and think about the system that you work in. **What makes it difficult for you to care for older patients following discharge and how do you overcome these challenges?** i.e. Everyday challenges (e.g. fulfilling follow ups, lack of info about med changes etc.)

As a practice what challenges do you face and how do you overcome them? e.g. inefficient system, staff shortages, periods of excessive workload.

How do you find out about the good and bad care that patients receive during their transitions home?

Older patients often don't feel very well prepared for their discharge – they don't fully understand what has happened to them, they have been deconditioned while in hospital etc. **Can you think of anything that hospital staff have done to effectively prepare older patients for discharge?** e.g. information giving, self-care, signposting, something to reduce deconditioning

---

Following a discharge home, how do you try and involve older patients and their families in their care?

- Is there anything else you think I need to know about how your team delivers safe care and / or involves patients during a transition from hospital to home?
- Who else should I be speaking to? (Within the team, wider teams, external healthcare teams)
- Anything I should specifically explore with secondary care?
